# Aqueous Manufacturing of Defect-Free Thick Multi-Layer NMC811 Electrodes

**DOI:** 10.3390/nano12030317

**Published:** 2022-01-19

**Authors:** Lukas Neidhart, Katja Fröhlich, Nicolas Eshraghi, Damian Cupid, Franz Winter, Marcus Jahn

**Affiliations:** 1 Electric Vehicle Technologies, Center for Low Emission Transport, Austrian Institute of Technology GmbH (AIT), Giefinggasse 2, 1210 Vienna, Austria; katja.froehlich@ait.ac.at (K.F.); nicolas.eshraghi@ait.ac.at (N.E.); damian.cupid@ait.ac.at (D.C.); 2 Institute of Chemical, Environmental and Bioscience Engineering, TU Wien, Getreidemarkt 9, 1060 Vienna, Austria; franz.winter@tuwien.ac.at

**Keywords:** multi-layer coating, aqueous electrode processing, NMC811, thick electrode

## Abstract

Manufacturing thick electrodes for Li-ion batteries is a challenging task to fulfill, but leads to higher energy densities inside the cell. Water-based processing even adds an extra level of complexity to the procedure. The focus of this work is to implement a multi-layered coating in an industrially relevant process, to overcome issues in electrode integrity and to enable high electrochemical performance. LiNi${}_{0.8}$Mn${}_{0.1}$Co${}_{0.1}$O${}_{2}$ (NMC811) was used as the active material to fabricate single- and multi-layered cathodes with areal capacities of 8.6 mA h cm^−2^. A detailed description of the manufacturing process is given to establish thick defect-free aqueous electrodes. Good inter-layer cohesion and adhesion to the current collector foil are achieved by multi-layering, as confirmed by optical analysis and peel testing. Furthermore, full cells were assembled and rate capability tests were performed. These tests show that by multi-layering, an increase in specific discharge capacity (e.g., 20.7% increase for C/10) can be established for all tested C-rates.

## 1. Introduction

The demand for Lithium-ion batteries (LIBs) used in xEVs has grown steadily over the past years alongside the interest to increase driving range and lower prices. A decrease in manufacturing cost combined with more environmentally friendly cell production methods are key aspects in battery research, helping e-mobility to establish itself as a day-to-day form of transportation.

One way of tackling the issue of high production costs is to exchange the organic solvent, used in state-of-the-art electrode slurry formulations, with water [1,2,3]. Commercially available cathodes are produced with polyvinylidene fluoride (PVDF) as the binder material. Unfortunately, due to its low solubility, PVDF is commonly dissolved in the high-priced N-methylpyrrolidone (NMP) organic solvent. Furthermore, expensive measures must be implemented to properly trap and filter the highly toxic NMP, which evaporates during the drying process of the electrodes. Binder materials, which are essential components in slurries, must fulfill several requirements. First, they are essential to guarantee good adhesion between the particle components as well as to the current collector foil. Uniform coatings rely on well dispersed slurries, which in turn depend on properly chosen binders. Moreover, the harsh environment inside the battery cell also requires chemically inert materials. Aqueous slurry preparation is advantageous, as it eliminates the utilization of both PVDF and NMP, leading to an overall reduction of material and production costs. Additionally, evaporation of water is not only more efficient but also needs up to 90% less energy during the drying process [4].

Although there are several advantages associated with replacing NMP by water, there are also some disadvantages due to the reactions of the cathode active materials with water. For example, cathode active materials are prone to react with water, causing extra complexity to the whole procedure. Li^+^ ions are leached from the lattice of the cathode active material, which is accompanied by the oxidation of the transition metal cations and loss of charge capacity. Furthermore, the leached Li^+^ ions induce a raise in the pH of the slurry, due to the formation of LiOH. However, oxide layers on aluminum current collectors are only stable up to a pH lower than 8.6 [5] before the Al foil corrodes. The corrosion is not only associated with an increase in interface resistance, but also with the evolution of hydrogen gas, which can result in large cavities inside the electrode layer. To prevent the development of defects during and after the coating process, several strategies exist to regulate pH values during slurry mixing. The usage of different acids, such as phosphoric acid [6,7,8], hydrochloric acid [9,10], poly acrylic acid [11] or acetic acid [12] is the most common method for maintaining the pH of the slurry within an adequate range between 4 and 8.6. The utilization of certain acids has another positive effect on the Li^+^ leaching mechanism. For example, adding phosphoric acid to the slurry can lead to the formation of an insoluble layer of phosphate compounds deposited on the surface of the active material particles, which suppress the ongoing leaching of transition metal cations from LiNi${}_{x}$Mn${}_{y}$Co${}_{z}$O${}_{2}$ (NMC) [13,14]. Zhu et al. showed that the viscosity of the slurry is also affected by the amount of acid added [12], as increasing the quantity of acetic acid leads to an increase in viscosity. Especially when targeting thick coatings, the viscosity has a significant influence on the film quality, which is influenced by the solid content of the slurry and binder characteristics. The solid loading of the slurry impacts the sedimentation rate of the coating and needs to be balanced carefully with solvent evaporation [15]. Binder concentration and molecular weight also affect the rheological behavior [15,16] and, consequently, the morphology of the electrode. In addition, a certain viscosity is necessary to maintain the desired thickness and shape of the electrode after the coating and drying process.

Increased energy densities can be realized by maximizing the ratio of active to inactive material [17,18,19]. Reducing separator and current collector materials leads not only to an increase in energy density, but also reduces the time required for cell assembly [19,20,21]. Energy density can further be increased by manufacturing thicker electrodes. However, thick electrodes tend to exhibit lower rate capabilities due to higher charge transfer resistance and blocked pore structures. The use of slurry additives and adjustment of coating parameters are widespread strategies to create crack free thick films. Controlling capillary pressures during the drying procedure by adding isopropyl alcohol to the slurry to regulate surface tensions [18] is one approach to reduce crack formation. Vapor grown graphite tubes as a replacement for carbon black can also help to minimize the amount of pinholes as recommended by Sahore et al. [22]. Beside maintaining mechanical integrity of the coating, a homogeneous distribution of the electrode material components are desirable, as accumulations of binder material can lead to unwanted side effects and diminished electrochemical performance. Binder migration is a large challenge during drying, thereby necessitating well optimized coating parameters. For example, the higher concentration of binder particles near the electrode-separator interface is a phenomenon that can be observed during the drying of thick coatings [23,24,25,26,27]. Light binder particles float on top of the layer due to a lack of liquid linkage between the surface and current collector. As a result, ion pathways are blocked and Li${}^{+}$ transport is impeded. The absence of binder at lower regions is also problematic for adhesion between coating and current collector [28].

This manuscript presents an alternative strategy to circumvent these difficulties in thick layer manufacturing by multi-layer coating. Applying multiple layers of coating on top of each other can help control film properties in a desired manner. Graded porosity was already investigated and presented as an interesting opportunity in the past [29,30,31,32,33]. Reduction in resistance, a more uniform overpotential distribution, and an increase in diffusivity of Li-ions are affected by establishing low porosity regions near the substrate and areas with high porosity in the vicinity of the separator [29,30,31,32]. However, implementing such a gradient is complicated as several subsequent calendaring steps are necessary, which reduces the expected positive effects [33]. Multi-layering also opens the doors to realize different material combinations inside the electrode. Blends of active materials can lead to higher ionic current densities [34], improvements against overcharging [35] and higher discharge rates [36]. For example, NMC442 cathodes [37] and graphite anodes that were fabricated [38] via dual layered slot-die coating show better electrochemical performance when the binder content of the top layer is reduced.

The purpose of this manuscript is to highlight the benefits of using multi-layering as a method to achieve high energy densities in cathode production. An industry oriented roll-to-roll (R2R) process was used to demonstrate the procedure using NMC811 as the cathode active material, which was coated in aqueous slurries. The resulting improvements in mechanical integrity and electrochemical performance are displayed. Due to its simplicity for industrial implementation, this technique is a stepping stone for the fabrication of unique electrode architectures. To the best of our knowledge, no defect free water-based NMC811 cathodes were established before with presented high areal capacity using the multi-layer approach.

## 2. Materials and Methods

Two different cathode coatings were performed—one thick single-layer (SL) of Ni-rich lithium nickel manganese cobalt oxide (NMC811) and one multi-layer (ML) coating with similar total electrode thickness. Each of the electrodes were analyzed with respect to their physical properties. In addition, coin cells were assembled to investigate their electrochemical performance.

### 2.1. Electrode Preparation

The cathode coatings consist of 92 wt% NMC811 powder (BASF SE, Ludwigshafen am Rhein, Germany; d_avg_ = 7.8 μm) as active material, 3 wt% carbon black (C-NERGY™ SUPER C65, TIMCAL Ltd., Bodio, Switzerland; d_avg_ = 37 nm) and 2 wt% artificial graphite (C-NERGY™ KS6L, TIMCAL Ltd.; d_50_ = 3.5 μm) as conducting agents, 2 wt% of CMC (WALOCEL™ CRT 2000 PA, DuPont de Nemours Inc., Wilmington, DE, USA) and 1 wt% of poly(meth)acrylate (PMA) (JSR SX8684(A)-64, JSR Micro NV, Leuven, Belgium) as binder. A total of 0.16 g of 1 M phosphoric acid (H_3_PO_4_) per g of NMC811 was added prior to addition of NMC to control the pH-value throughout the mixing process. All solid components used in this study were dried over night at 105 ${}^{{}^{\circ}}C$.

Slurry mixing was done in a 250 mL container using a dissolver (DISPERMAT CV3-PLUS, VMA GETZMANN GMBH, Reichsdorf, Germany). Under constant stirring at 200 rpm, carbon black (CB) and artificial graphite (KS6L) were added to a 2 wt% CMC solution, following 10 min of stirring at 2000 rpm. H_3_PO_4_ was added, followed by another 2 min of stirring. NMC811 was added at 3000 rpm for 8 min. Finally, PMA was added at maximum 500 rpm stirring for 2 min to keep material integrity, terminating in a solid content of 60%. The pH was measured (SevenCompact S210, Mettler Toledo, Columbus, OH, USA) to monitor that values are below 8.6 before coating.

Electrode casting was performed continuously on a R2R coating machine (SC 30, COATEMA Coating Machinery GmbH, Dormagen, Germany) on 22 μm aluminum foil (Norsk Hydro ASA, Oslo, Norway). Three consecutively arranged drying chambers were set to temperatures of 45, 55 and 50 ${}^{{}^{\circ}}C$. The flow rates at the air inlet and outlet valves were set to 70 $m^{3}$ h^−1^ and 98 $m^{3}$ h^−1^, respectively. The coating speed was fixed at 0.3 m min^−1^. The gap size of the coating knife was set to 550 μm for the thick single-layer, whereas for the multi-layer coatings, the wet thickness of the bottom layer was set to 250 μm and of the top layer was set to 330 μm. The bottom layer was fully dried before adding the second coating. To remove residual water, all samples were dried at 80 ${}^{{}^{\circ}}C$ under vacuum for 12 h. Afterwards, the porosity ϵ of the electrodes was calculated by using Equations (1)–(3), where $\rho_{c}$, $m_{c}$ and $V_{c}$ are the density, mass, and volume of the coating, respectively and $\rho_{ph}$ is the sum of all bulk densities $\rho_{i}$ with respect to their share $p_{i}$ of the coating:

(1) $$\rho_{c}=\frac{m_{c}}{V_{c}},$$

(2) $$\rho_{ph}=\sum_{i}p_{i}\rho_{i},$$

(3) $$\epsilon =1-\frac{\rho_{c}}{\rho_{ph}}.$$

Dried electrode sheets were calendared at 55 ${}^{{}^{\circ}}C$ roll temperature (GK 300L, SAUERESSIG Group, Vreden, Germany) to a target porosity of 40% and a final thickness of approximately 205 μm including the current collector foil.

In the full cell investigations, anodes fabricated at the pilot line facilities were used as standard counter electrodes. All anodes used in this study were made of 90 wt% high energy density graphite (HED graphite 918-A2, Targray Technology International Inc, Kirkland, QC, Canada; d_50_ = 14.93 μm), 4 wt% carbon black (C-NERGY™ SUPER C65, TIMCAL Ltd.) and 6 wt% PVDF (Solef^®^ PVDF, Solvay SA, Brussels, Belgium). The slurry was prepared in a planetary mixer (HIVIS DISPER MIX Model 3D-2, PRIMIX Corporation, Awaji-shi, Japan). First the active material and the carbon black were mixed together. PVDF, which was dissolved in an 8 wt% solution of NMP, was added and the slurry was mixed with increasing rotational speed. Additional NMP was added to dilute to a final solid content of 50%. The anode was coated on 11 μm copper foil (Carl Schlenk AG, Roth, Germany) with a wet thickness of 560 μm and afterwards dried and calendared to a porosity of 38%. A practical specific capacity for the graphite active material was assumed to be 350 mA h g^−1^. An areal capacity of 9.5 mA h cm^−2^ for graphite anodes leads to a N/P ratio of 1.1.

Coating parameters and electrode materials are changed as little as necessary compared to a standardized manufacturing process to demonstrate the feasibility of the water based multi-layering technique to an existing production procedure.

### 2.2. Cell Assembly and Electrochemical Analysis

For coin cell tests, cathodes and anodes were cut into discs of 1.5 cm and 1.6 cm diameter, respectively. The anodes were dried at 120 ${}^{{}^{\circ}}C$ and the cathodes at 80 ${}^{{}^{\circ}}C$ under vacuum for 12 h before being transferred in vacuum into an Ar-filled glove box (O_2_ < 0.1 ppm, H_2_O < 0.1 ppm) (LabMaster Glove Box MB200-G, MBRAUN, Garching, Germany). Then, 2032 coin cells (CR2032) were assembled with 1.1 mm springs, 1.5 mm spacers and a Celgard 2500 separator. In total 150 μL of the electrolyte were added before crimping (MSK-110 Hydraulic Crimping Machine, MTI Corporation, Richmond, CA, USA) at 60 bar pressure.

After a resting time of 4 h, cycling tests were performed on an Arbin BT-21084, assuming 200 mA h g^−1^ capacity for the cathode active material. Two cycles with a C-rate of C/20 were carried out for formation, followed by five preconditioning cycles at C/10. The discharge capacity for further tests was adjusted to be in-line with the capacity of the third C/10 cycle. Thus, further tests were carried out with a specific capacity of 146 mA h g^−1^ and 177 mA h g^−1^ for SL and ML electrodes, respectively. Constant current constant voltage (CCCV) rate capability tests with symmetric charge/discharge rates of C/5, C/2, 1C and C/10 were also conducted in a voltage window of 3 to 4.2 V. To determine the long term cycling behavior, 50 cycles at a C-rate of C/5 were performed for both cell types.

Electrochemical impedance spectroscopy (EIS) was used to investigate the influence of multi-layering on the kinetics during cycling within the cell. Measurements were performed at 4.2 V/100% state of charge (SOC) during the two formation cycles at a C-rate of C/20. Potentiostatic electrochemical impedance was measured between 20 kHz and 100 mHz with a voltage amplitude of 10 mV on a Biologic MPG-2 instrument. Cyclic voltammetry (CV) measurements were performed to compare reduction and oxidation processes for SL and ML electrodes in half cell configurations. The applied range of potential was set between 2.8 V and 4.5 V with a scan rate of 0.05 mV s^−1^ on an Arbin BT-21084.

### 2.3. Slurry and Electrode Properties

Surface topologies and cross-sectional imaging of the electrode samples were carried out via a digital microscope (VHX7000, Keyence Corporation, Osaka, Japan), to evaluate coating homogeneity and investigate inter-layer transitions of the multi-layered electrodes. The electrode samples were also investigated via scanning electron microscopy (SEM) at an electron acceleration voltage of 5 kV (SUPRA 40, Carl Zeiss AG, Oberkochen, Germany). Furthermore, the adhesive strength of the coating to the current collector foil was characterized using a 180° peel test (EKM-5KN, Jinan Marxtest Technology Co., Ltd., Jinan, China).

Viscosity measurements were performed under ambient conditions (DV-II+Pro Viscometer, AMETEK Brookfield, Middleboro, MA, USA), and dynamic viscosity was recorded with respect to increasing shear rates between 0 and 14 s^−1^.

## 3. Results and Discussion

### 3.1. Characterization of Slurries and Electrodes

Since the pH of the slurry has a huge impact on the process, the pH evolution was carefully monitored to stay below the recommended coating limit of 8.6 [5] to suppress possible corrosion of the Al substrate. This is also important with regard to the emergence of agglomerations within the slurry reported to occur when the pH of 10 is exceeded in the slurry mixing process as described above in Section 2.1. Viscosity measurements showed a shear thinning behavior of 18 Pa s at zero shear rate and 4 Pa s at the shear rate applied during the coating process. Porosity and areal capacity were calculated based on the measured thicknesses and mass loadings in SL and ML electrodes. The values of the measured parameters are given in Table 1. Coating thicknesses are slightly higher than 200 μm for both types of electrodes. The as-coated porosity values of the SL and ML samples are calculated to be almost identical with each other, attaining values of 52.7 and 52.6% for the SL and ML electrodes, respectively. Both coatings show the same areal capacity of 8.6 mA h cm^−2^, which was calculated assuming a 200 mA h g^−1^ specific capacity for the NMC811 active material. The lower layer constitutes a 43% share of the overall bi-layer electrode thickness. On average, a porosity of 59.3% was calculated for the base layer with an areal capacity of 3.1 mA h cm^−2^.

**Figure 1:**
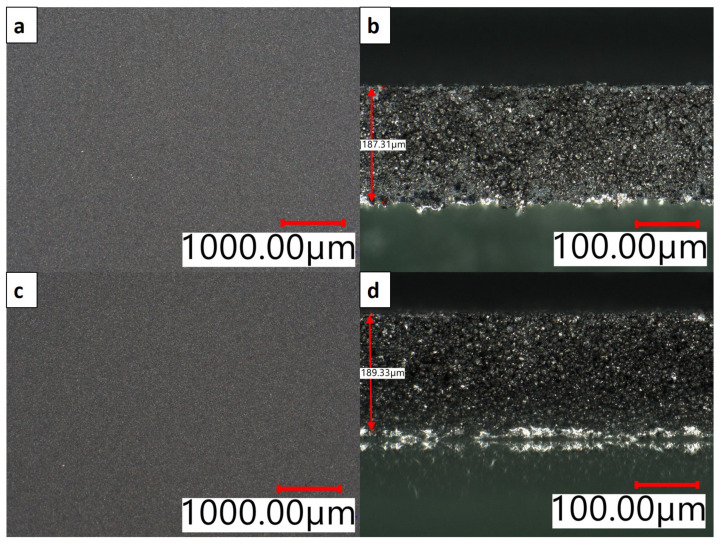
Digital microscope images of (**a**) SL top-view, (**b**) SL cross-section, (**c**) ML top-view, (**d**) ML cross-section after drying in air.

Investigating the surface of each coating, it is evident that a continuous film without defects of any kind was fabricated (Figure 1a,c). Even before calendaring, a smooth homogeneous surface was present in both samples. This is a huge improvement considering the high thickness and aqueous processing of the electrode [18,22].

**Figure 2:**
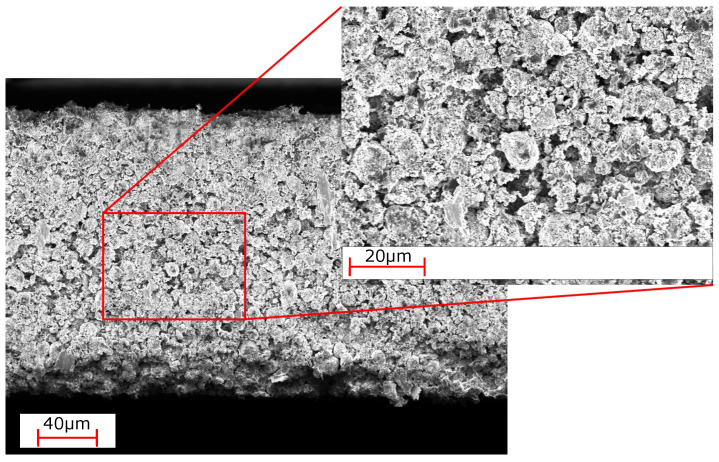
SEM image of the transition area from top to bottom layer of a multi-layer coated sample.

Analysis of cross sectional images taken via digital microscope are shown in Figure 1b,d for single- and multi-layer electrodes, respectively. Both samples are determined to have a thickness of 190 μm after calendaring to 40% porosity, verifying the measurements via a thickness gauge. Slightly inhomogeneous distribution of lighter gray particles is noticeable for SL samples, but overall no severe irregularities, such as entrapped air, cracks, or delamination from the substrate are recognizable. No distinct transition line between top and bottom layer is visible for the multi-layer sample, which is evidence of a good mechanical connection of both layers. SEM images of the multi-layer sample are displayed in Figure 2. Merging of the layers is visible in agreement with the analysis via digital microscope. Images of higher magnification reflect an intertwining behavior of the secondary NMC811 particles of the top and bottom layers, which is necessary for strong cohesion. The indistinguishability of the two layers can also be accredited to the slight “dissolution” of the surface of the bottom layer into the freshly applied electrode slurry of the upper layer during coating. Furthermore, the surface film of the prime coating absorbs the slurry of the second coating forming a diffuse transition layer. This is a key aspect in guaranteeing good mechanical linkage of both layers.

**Figure 3:**
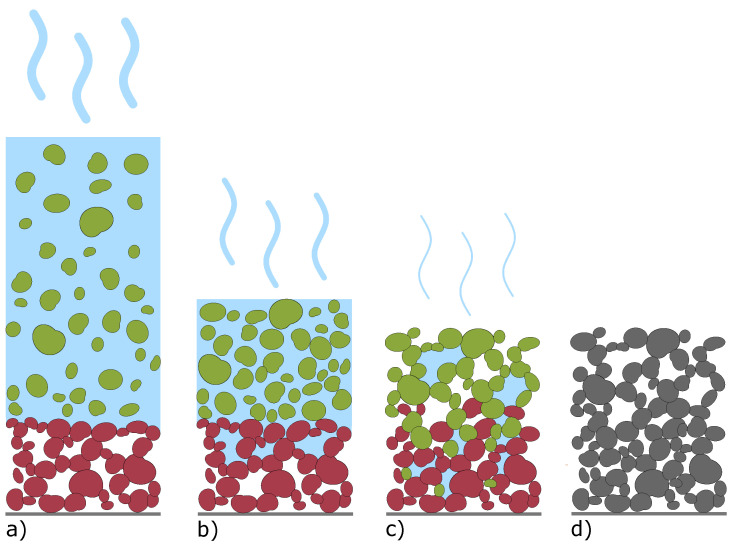
Visualization of the trickling behaviour after the second coating is applied. Particles in red and green, of the first and second layer, respectively. (**a**) situation right after coating the second layer, (**b**) sedimentation of particles during water evaporation, (**c**) mixing of second and first layer particles creating a diffuse interface, (**d**) coating after full solvent evaporation with higher porosity close to the current collector.

The significantly higher porosity of the lower layer also implies either a change in porosity of the layer itself due to the coating of the upper layer, or a porosity gradient with remarkably higher porosity in the vicinity of the current collector. Assuming no porosity transition of the base after the second coating, the porosity of the top layer should be 47.5%. According to both Qi et al. [30] and Fang et al. [31] this lower porosity of the top layer would lead to a decreased cell performance as they suggest an electrode design with an opposite porosity gradient (higher porosity at the top of the electrode with decreasing porosity in the direction of the current collector) to reduce electrode resistance and enhance performance at high C-rates by increasing Li-ion diffusivity. However, the higher observed porosity of the upper layer, as well as the observed bubbles which develop after the second coating is applied, imply that cavities inside the base layer are filled with applied slurry. Figure 3 illustrates this mechanism. First, the slurry of the second coating comes in contact with the low porosity base layer (Figure 3a). As the water evaporates, porosity decreases in the top layer (Figure 3b) and the particles from the top layer migrate, and occupy the cavities in the bottom layer. This causes a mixing of both coatings (Figure 3c) and results in an increase in porosity of the initial layer (Figure 3d). This influences the porosity gradient to generate lower porosity values near the substrate foil and higher porosity values closer to the coating surface.

**Figure 4:**
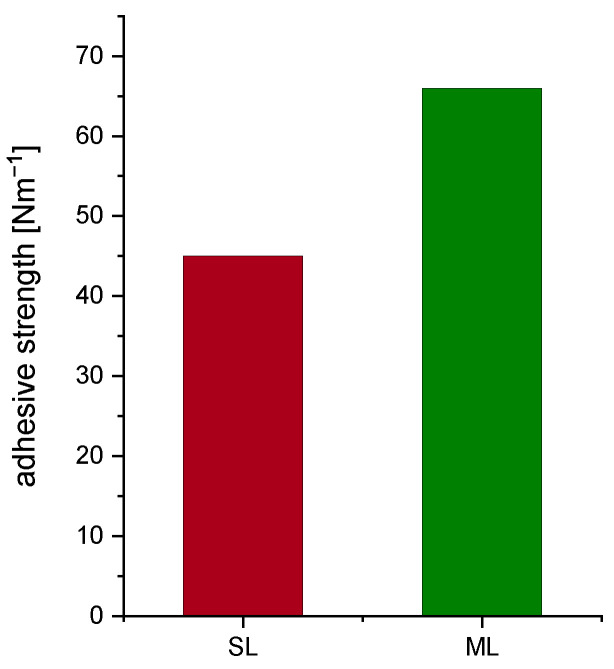
Comparison of the adhesive strength for SL and ML electrodes.

The results of the peel testing measurements confirm significant improvement following the multi-layer approach. In comparison to SL samples with ∼45 Nm^−1^, ML coatings show values of ∼66 Nm^−1^ (Figure 4). Therefore, on account of the second coating, an increase of approximately 45% in adhesion can be achieved. The reason for this vast enhancement is due to more homogeneously distributed binder particles, which is enhanced by the multi-layer processing.

### 3.2. Electrochemical Performance of Cells

**Figure 5:**
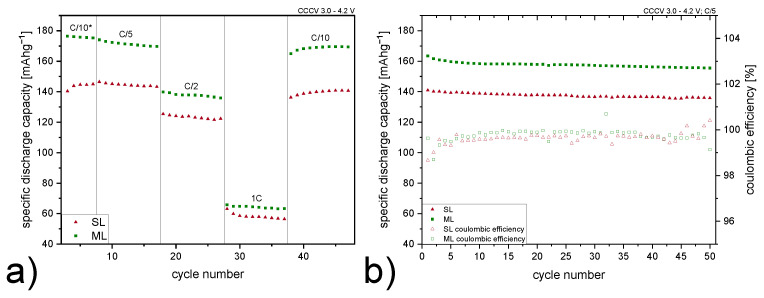
(**a**) Rate capability tests for single-layer (SL, red) and multi-layer (ML, green) electrodes with C-rates of C/10, C/5, C/2 and 1C. Cycles for preconditioning are labeled with C/10*. (**b**) Long term cycling at C/5 and coulombic efficiency of both electrode types.

Rate capability tests were performed to assess the electrochemical performance of both cell configurations. Results of statistically significant cells are displayed for clear visualization (Figure 5a). Formation cycles are not presented in the graph. It is worth mentioning, that after preconditioning at C/10 for 5 cycles, the C-rate was adjusted according to the measured cell discharge capacity of cycle number 3 (these preconditioning cycles are labels as C/10* in Figure 5a). Therefore, all cathodes are exposed to the same C-rate independent of the actual specific capacity of the cathode after the preconditioning cycles. In accordance, the ML cells show higher specific discharge capacities throughout all the tests, even though the same C-rates are applied. Especially for low current densities, cells with ML cathodes outperform the SL samples by over 20%, which is a remarkable enhancement considering that both electrodes are fabricated using the same materials. Therefore, changing the coating procedure has a positive impact on material distribution inside the coating. The beneficial effect of multi-layering is even more pronounced for lower C-rates. For thick electrodes, limiting effects on Li^+^ diffusion are evident at high current densities, and resulting losses in discharge capacity can not be compensated fully by multi-layering. Therefore the difference in capacity is slightly less distinct for 1C. Approximate improvements of the discharge capacities are listed in Table 2. When returning back to C/10, both cell configurations show initial high capacities, implying that cycling at high rates is not detrimental to their stability. Long term cycling tests show no significant decrease in specific discharge capacity after 50 cycles at C/5 for SL and ML electrodes (Figure 5b). Moreover, the coulombic efficiency for both SL and ML electrodes is determined to be close to 100%.

**Figure 6:**
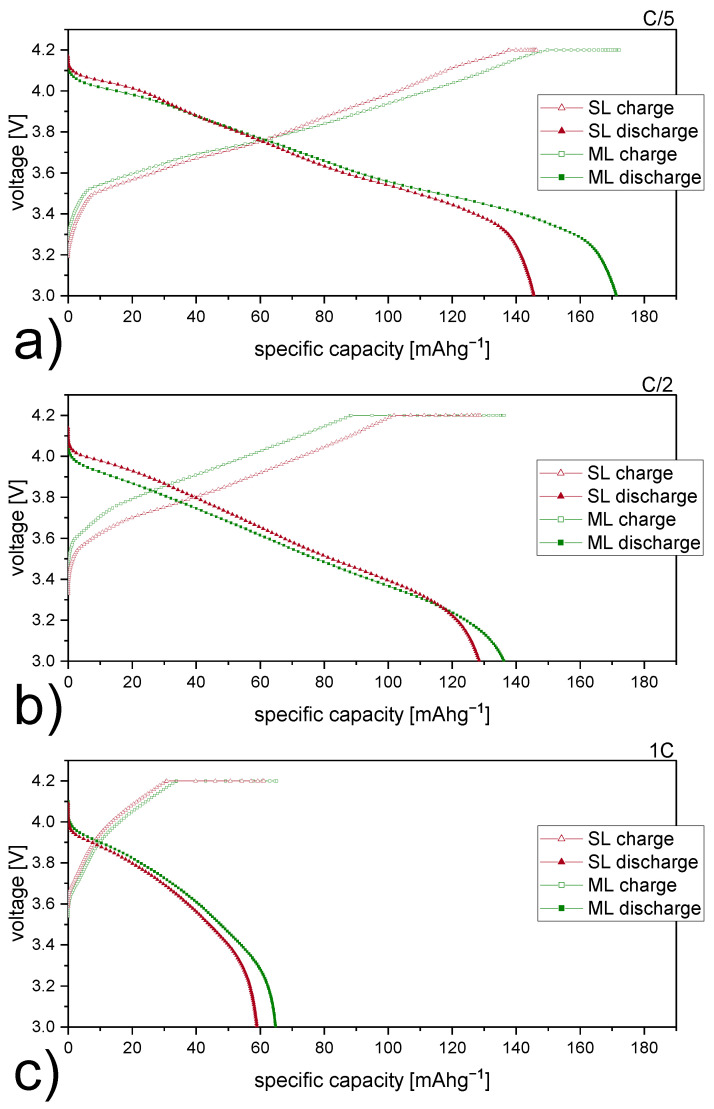
Voltage profiles of single-layer (SL, red) and multi-layer (ML, green) electrodes at (**a**) C/5, (**b**) C/2, and (**c**) 1C.

Specific capacities of representative charge and discharge cycles are selected for comparison at each C-rate and are plotted in Figure 6 against applied voltages. The specific discharge capacity data as a function of C-rate are also given in Table 3. SL cells show values of 146 mA h g^−1^, 128 mA h g^−1^ and 59 mA h g^−1^ for C/5, C/2, and 1C, respectively. ML cells have the overall highest specific discharge capacity of 171 mA h g^−1^ at C/5, whereas at C/2 and 1C, capacities of 136 mA h g^−1^ and 65 mA h g^−1^ are measured. Substantial differences in discharge capacity between the two types of electrodes are present for low current densities. For single layers only the region close to the electrode/separator interface is electrochemically active, since Li ion diffusion lengths are not sufficient enough to reach underlying areas [39]. However, in ML electrodes, areas located closer to the current collector foil also contribute to specific discharge capacity. Figure 6 shows the voltage-capacity curves of the SL and ML electrodes at C-rates of C/5 (a), C/2 (b) and 1C (c). All of them show a sloping profile, which is characteristic for NMC cathode active materials. In all cases, the charge and discharge capacities of the cells with the ML electrodes are larger than those for the SL electrodes, despite the fact that all electrodes have the same mass of active material. At high current densities, the benefits of multi-layering on Li-ion diffusion compensate for the drawbacks accompanied by electrode thickness itself [40]. Li-ion mobility definitely poses the most difficulties for thick electrodes to compete at high C-rates.

The potentiostatic contribution to the specific charge capacity is represented by the plateau at 4.2 V in Figure 6. Its length is directly proportional to the resistance inside the electrode during charge at constant voltage. The presence of multiple layers causes additional interfacial resistances within ML coated electrodes. Especially at C/5 (Figure 6a), a distinct difference in potentiostatic specific charge capacity is noticeable. At low C-rate in particular, a great part of the capacity is reached due to a elongated constant voltage step during charge. Nevertheless, it is worth mentioning that at C/5 higher specific charge capacities are already reached before the constant voltage step takes place.

**Figure 7:**
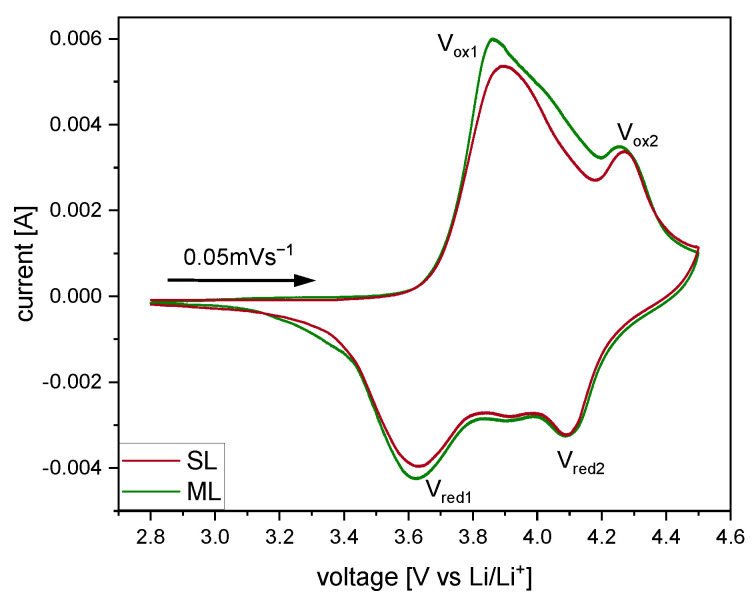
Cyclic voltammograms of single-layer (SL, red) and multi-layer (ML, green) electrodes between 2.8 and 4.5 V, with a scan rate of 0.05 mV s^−1^.

The cyclic voltammograms shown in Figure 7 were recorded to compare the reduction and oxidation reactions of SL and ML electrodes during cycling. Both electrode types show no peak at 3 V, indicating the absence of Mn^3+^ [41]. Two pairs of oxidation/reduction peaks were observed for each sample. The small reduction peaks for SL and ML at around 3.9 V are not visible in the CV during oxidation due to overlapping with the lower voltage reduction peaks. Table 4 displays the potentials of oxidation/reduction peaks and corresponding polarizations for each electrode type. SL cells show oxidation peaks at V_ox1_ = 3.90 V and V_ox2_ = 4.27 V and reduction peaks at V_red1_ = 3.63 V and V_red2_ = 4.09 V, with a polarization of ΔV_1_ = 0.27 V and ΔV_1_ = 0.18 V respectively. The polarizations for ML cells are ΔV_1_ = 0.24 V for peaks at V_ox1_ = 3.86 V and V_red1_ = 3.62 V and ΔV_1_ = 0.16 V for V_ox2_ = 4.25 V and V_red2_ = 4.09 V. Comparing both electrode types, no significant differences in peak position and polarization are noticeable. However, ML samples show a larger area underneath the curve, which is in direct proportion to the capacity of the cell and is in accordance with the capacity difference shown in the rate capability test mentioned above. Besides the higher capacities, no obvious difference of the reaction kinetics were observed via CV measurements.

**Figure 8:**
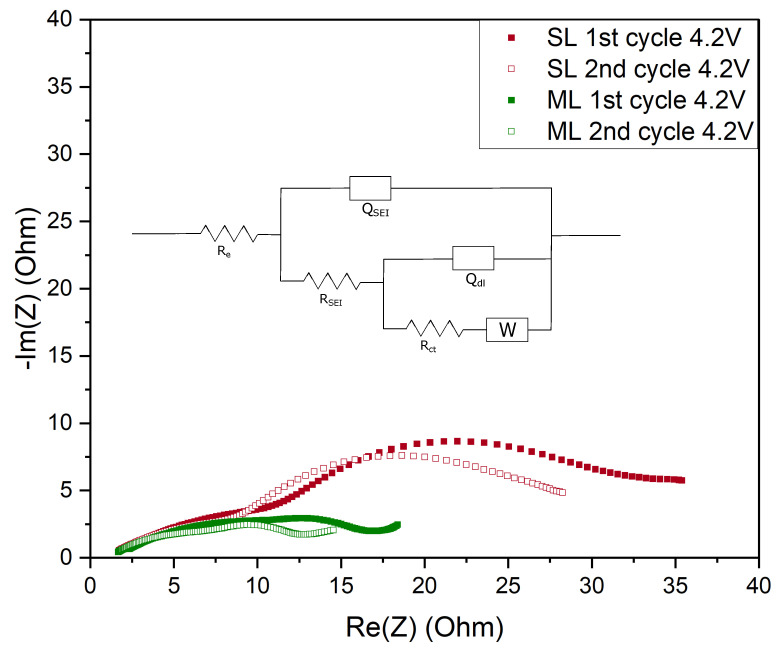
Equivalent circuit used to fit the measured impedance spectra and Nyquist plots of 1st and 2nd cycle at 4.2 V for single and multi-layer cathodes.

EIS measurements were performed to help describe the transfer phenomena inside the cells during the formation cycles. The equivalent circuit used for the fitting is displayed in Figure 8. R_e_ corresponds to the bulk resistance resulting from the cell components (current collector, separator) and the electrolyte. The resistance contribution from the solid electrolyte interphase (SEI) formed on the graphite anode is fitted using R_SEI_ and a constant phase element Q_SEI_, expressing its behavior as the non ideal capacitor. The charge transfer resistance R_ct_ and the double-layer capacitance represented by Q_dl_ show the contribution of charge transfer behaviour between the electrolyte and the electrode. The diffusion at low frequencies is represented by the Warburg element (W).

**Figure 9:**
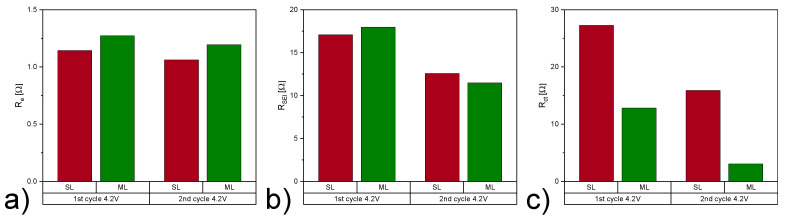
Comparison of resistance contributions (**a**) R_e_, (**b**) R_SEI_, and (**c**) R_ct_ for SL and ML cells during the 1st and 2nd cycle at 4.2 V.

Figure 8 shows Nyquist plots of SL and ML cells during the first and second cycle at 4.2 V. The intercept of Re(Z) at the high frequency region shows similar values in R_e_ for both samples, as shown in Table 5 and Figure 9a. This is expected, since all components apart from the cathodes are identical for each cell. The high to medium frequency semicircle shows the contribution of SEI formed during the first cycle on the anode side. At different stages of charge and discharge for the SL and ML electrodes the fitted data is comparable, highlighting the fact that the developed cathode in this work has no negative impact on the development of the SEI and its stability.

The mid to low frequency semicircle illustrates the charge transfer process, where a significant difference was observed between the charge transfer resistance of SL and ML electrodes. The higher specific discharge capacity, as shown in Figure 6, for the multi-layer cells, is attributed to the lower R_ct_ value. Heubner et al. [42] investigated the influence of electrode porosity and thickness on cell performance, with respect to limiting processes. They conclude that among other effects, decreased porosity leads to an increase in charge transfer resistance. Available areas for charge transfer reactions are reduced due to an increase in contacts between particles and particles with current collector foil and a reduction in the total contact area with the particles and the electrolyte [42]. The pore size distribution in the ML electrode facilitates access of Li-ions from the electrolyte in the whole multi-layer electrode and thus lowering the R_ct_. Figure 9 displays the acquired data for both electrode types and shows that a lower R_ct_ is achieved and a stable performance is established throughout cycling. The short inclined line at low frequencies represents the Li diffusion into the active material. Similar values for SL and ML samples lead to the conclusion that multi-layering does not negatively influence the diffusion of the Li-ions in the electrode and the mass transport controlled region of the cathode.

The advantage of better adhesion in ML samples is not expected to be fully realized in coin cell configurations, since the high pressure inside the cells suppresses the delamination. Therefore, we anticipate a more pronounced benefit in pouch-cell configurations. Considering all electrochemical results, more homogeneous binder distribution and the resulting increase in charge transfer leads to higher discharge capacities in ML cells. In addition, better electrochemical performance roots from a porosity gradient generated inside the cathode—as suggested in literature [30,31].

## 4. Conclusions

Aqueous fabrication of thick NMC811 electrodes was performed using an industrially relevant coating procedure. pH regulation with phosphoric acid and optimized coating parameters lead to smooth thick cathode films with superior areal capacities of 8.6 mA h cm^−2^. It was demonstrated that multi-layer electrodes of equal active material loading can be fabricated in a subsequent tape casting procedure. The absence of morphological defects was confirmed for the presented manufacturing technique. Both electrodes exhibit sufficient mechanical integrity to be processed on a standard roll-to-roll system. Multi-layer casted cathodes show improved adhesion to the Al current collector, exemplified by a 45% higher adhesion strength for the ML electrodes compared to SL. In addition, optical inspection revealed a diffuse boundary between the separately coated layers, confirming good intra-layer adherence. ML electrodes show an improved electrochemical performance during rate capability tests compared to SL electrodes, expressed by higher specific discharge capacity for all investigated C-rates. It is worth mentioning that, especially for low current densities, improvements of over 20% are measured. This occurs due to more evenly distributed material inside the cathode coating and a possible gradient in coating porosity. All findings emphasize the potential of using multi-layering as a technique to gain energy density through increased electrode thickness. This technique can be easily adapted to R2R processing and a variety of electrode fabrication applications on an industrial scale. This study shows that by multi-layering, great improvements in electrochemical performance and mechanical stability can be achieved within aqueous fabrication of high energy density cathodes. It also opens up new possibilities for multiple design options by implementing various material components and properties into the electrode. The 3D architectures can be easily integrated within coatings in a consecutive large scale casting method. Future work will investigate cell performance for different material gradients, such as binder, conducting agents, and particle size in a two or more layer electrode design. Opportunities to decrease tortuosity and thereby shorten Li-ion pathways can be realized by varying film composition through multi-layer coating. This will be the subject of future investigations.

## Figures and Tables

**Table 1:** Coating parameters after coating and drying.

Electrode Type	Total Thickness [μm]	Porosity [%]	Areal Capacity [mA h/cm^2^]
Single-layer	202 ± 2	52.7 ± 0.3	8.6 ± 0.1
Multi-layer	208 ± 4	52.6 ± 0.2	8.6 ± 0.2
Bottom layer	89 ± 5	59.3 ± 0.9	3.1 ± 0.0

**Table 2:** Increase of discharge capacities by multi-layering, compared to single-layer cathodes.

	C/10*	C/5	C/2	1C	C/10
Improvement [%]	23.3	18.6	11.7	10.3	20.7

**Table 3:** Comparison of specific discharge capacities for different C-rates for SL and ML cells.

C-Rate	Electrode Type	Specific Discharge Capacity [mA h g^−1^]
C/5	SL	146
	ML	171
C/2	SL	128
	ML	136
1C	SL	59
	ML	65

**Table 4:** Comparison of anodic and cathodic peak positions and corresponding polarization from CV measurements for SL and ML half cells.

Electrode Type	V_ox1_ [v]	V_red1_ [v]	ΔV_1_ [v]	V_ox2_ [v]	V_red2_ [v]	ΔV_2_ [v]
SL	3.90	3.63	0.27	4.27	4.09	0.18
ML	3.86	3.62	0.24	4.25	4.09	0.16

**Table 5:** Impedance parameters derived from the fitting of equivalent circuit models for SL and ML electrodes.

	Electrode Type	R_e_ [Ω]	R_SEI_ [Ω]	R_ct_ [Ω]	Q_SEI_ [mF]	α _SEI_	Q_dl_ [mF]	α_dl_A	χ ^2^
1st cycle 4.2 V	SL	1.14	17.08	27.29	4.90	0.45	2.85	1	0.006
	ML	1.27	17.97	12.81	5.91	0.45	7.36	0.99	0.009
2nd cycle 4.2 V	SL	1.06	12.58	15.88	6.50	0.45	4.23	0.98	0.004
	ML	1.19	11.48	3.05	6.48	0.45	7.90	1	0.005

## Data Availability

The data presented in this study are available on request from the corresponding author.

## Abbreviations

The following abbreviations are used in this manuscript:

CB	carbon black
CCCV	constant current constant voltage
CMC	carboxymethyl cellulose
CV	cyclic voltammetry
EIS	electrochemical impedance spectroscopy
ML	multi-layer
NMC	lithium nickel manganese cobalt oxide
NMP	N-Methyl-2-pyrrolidone
N/P ratio	negative/positive capacity ratio
PMA	poly(methyl)acylate
PVDF	polyvinylidene fluoride
R2R	roll-to-roll
SEI	solid electrolyte interphase
SEM	scanning electron microscopy
SL	single-layer
SOC	state of charge
